# Development, structure, and implementation of cardiometabolic clinics in the United States, with a focus on comprehensive patient care

**DOI:** 10.1016/j.ajpc.2026.101519

**Published:** 2026-03-01

**Authors:** Jodeanna Sweeney, Jason Liu, Gregory Warren, Alexander J. Blood

**Affiliations:** a Division of Bariatric Surgery, School of Medicine, Oregon Health & Science University, 3181 S.W. Sam Jackson Park Road, Portland, OR 97239, USA; b John Muir Health Walnut Creek Medical Center, 1450 Treat Blvd Ste #220B, Walnut Creek, CA 94597, USA; c Axene Health Partners, LLC, Littleton, CO, USA; d Heart & Vascular Center, Brigham and Women’s Hospital, 70 Francis St, Boston, MA 02115, USA

**Keywords:** Cardiometabolic syndrome, Cardiometabolic risk factors, Metabolic syndrome, Primary prevention, Secondary prevention, Cardiovascular disease, Preventive health programs

## Abstract

Metabolic syndrome and associated cardiometabolic risk factors affect over one‑third of US adults, increasing the risk for atherosclerotic cardiovascular disease, type 2 diabetes, and chronic kidney disease. Recently termed cardiovascular-kidney-metabolic syndrome, the syndrome contributes to substantial clinical and economic burden. Despite guideline recommendations, affected patients who have multimorbidity often receive suboptimal, fragmented care. Cardiometabolic clinics (CMCs) comprise a multidisciplinary team that deliver comprehensive, preventive care to patients with cardiometabolic risk factors and diseases. This review outlines key considerations for the development, structure, and implementation of CMCs in the US. Initial steps include developing a financial model to demonstrate potential return on investment, defining the target patient population, establishing care delivery and operational plans, such as cost coverage evaluations, and leveraging digital solutions to improve access and engagement. The proposed CMC structure comprises a core team of a navigator and specialist in cardiometabolic diseases, based on two US-based models: the comprehensive care model and Cardiometabolic Center Alliance – Cardiometabolic Center of Excellence model. Training the team across cardiometabolic conditions is essential. Once established, demonstrating a positive return on investment can support expanding the team to additional specialties. Scalability remains crucial due to the rising prevalence of cardiovascular-kidney-metabolic syndrome. Studies show CMCs increase uptake of guideline-directed medical therapies and improve cardiometabolic outcomes, although larger, long-term studies are needed. Benefits also extend to healthcare providers, including improved communication, greater convenience, and reduced redundant tests and visits, supporting cost savings. CMCs can potentially reduce the clinical and economic burden of cardiovascular-kidney-metabolic syndrome in the US.

**List of abbreviations**
ASCVD: atherosclerotic cardiovascular disease
CKD: chronic kidney disease
CKM: cardiovascular-kidney-metabolic
CMC: cardiometabolic clinic
CME: Continuing Medical Education
CVD: cardiovascular disease
GDMT: guideline-directed medical therapy
MetS: metabolic syndrome
ROI: return on investment
T2D: type 2 diabetes
US: United States
VOI: value on investment

## 1 Introduction

Metabolic syndrome (MetS) and associated cardiometabolic risk factors are highly prevalent across the globe [1], with the syndrome occurring in more than one-third of adults in the United States (US) [2,3]. MetS encompasses a cluster of risk factors, including elevated waist circumference, triglycerides, and blood pressure; insulin resistance; and reduced high‑density lipoprotein cholesterol [4,5]. The presence of these cardiometabolic risk factors puts individuals at a high risk for developing atherosclerotic cardiovascular disease (ASCVD), type 2 diabetes (T2D), and chronic kidney disease (CKD) [[4], [5], [6]]. In recognition of the link between these risk factors and poor health outcomes, the American Heart Association recommends adopting the term “cardiovascular-kidney-metabolic (CKM) syndrome” [6]. CKM syndrome is classified into stages: stage 0 is characterized by the absence of risk factors, stage 1 by excess or dysfunctional adiposity, stage 2 by metabolic risk factors and CKD, stage 3 by subclinical cardiovascular disease (CVD) in CKM, and stage 4 by clinical CVD in CKM [6]. The syndrome leads to significant morbidity and mortality, with CVD remaining the leading cause of disability and death both in the US and worldwide [[7], [8], [9]]. The syndrome also imparts a substantial economic burden, with healthcare utilization and medical costs increasing incrementally with each component present [10,11].

Guidelines recommend screening for cardiometabolic components and providing lifestyle and/or pharmacologic therapies tailored to a patient’s risk factor profile and preferences to ultimately delay or prevent the onset of disease [6,[12], [13], [14], [15], [16], [17]]. Preventing excess and/or dysfunctional adiposity, including obesity, is a central focus of CKM prevention due to its pathological role in the development of other metabolic risk factors and diseases, such as hypertriglyceridemia, hypertension, and T2D [6,17]. Since almost a decade, obesity has been recognized as a chronic condition termed “adiposity-based chronic disease”, which captures all aspects of the disease, from excess or abnormal adiposity to obesity-related complications, such as ASCVD, T2D, and CKD [17]. In turn, the American Association of Clinical Endocrinology consensus statement proposes principles for person-centered and complication-centric management that involves individualized lifestyle, pharmacologic, and/or surgical options (e.g., bariatric surgery) [17]. This proposal overlaps with guidelines that recommend lifestyle and disease-specific therapies to manage cardiometabolic risk factors in patients with ASCVD and T2D, using a shared decision-making approach to ensure individuals’ needs and preferences are taken into consideration [6,13,15,16]. Management therefore requires an interdisciplinary approach to address each cardiometabolic component as well as the various aspects of prevention and treatment, ideally involving a multidisciplinary team of primary care providers and specialists [6,13,16,17]. However, such interdisciplinary strategies are lacking in routine clinical practice. Healthcare providers often manage these patients with multimorbidity in silos, leading to challenges in delivering guideline-directed medical therapies (GDMTs) and patients receiving suboptimal, fragmented care [[18], [19], [20]]. Furthermore, despite guidelines recommending pharmacologic and/or surgical options as an adjunct to lifestyle interventions in obesity and CKM management, limited or absent insurance coverage and high out-of-pocket expenses remain major barriers to evidence-based care [21,22]. Hence, there is a need for comprehensive management strategies to improve patient outcomes, reduce the burden on healthcare providers, and minimize the associated costs. Cardiometabolic clinics (CMCs) have the potential to address these issues.

CMCs comprise a multidisciplinary team of professionals such as physicians, advanced practice providers, nurses, pharmacists, nutritionists, psychologists, and physical therapists that provide comprehensive care to patients with cardiometabolic risk factors [[23], [24], [25]]. Global and regional CMC models have been proposed; however, national and local needs in the US must be considered to ensure CMCs can be practically set up within the healthcare system in a manner that supports both patients and healthcare providers [[24], [25], [26]]. The aim of this review is to provide an overview of the development, structure, and implementation of CMCs in the US, by outlining practical considerations and potential hurdles and benefits of CMCs for patients, healthcare providers, and payers.

## 2 Development of CMCs

### 2.1 Financial justification

An important consideration when planning the development of a CMC in the US is the need for financial justification. Cost-effective or cost-saving cardiovascular prevention programs are a priority in the US, where private, commercial, and public payers carefully consider both the economic and societal value of covering programs [27,28]. Financial justification is particularly important given the lack of published evidence on the cost-effectiveness of CMCs in the US. Moreover, modelling studies across Europe have shown contrasting results as to whether primary care-based cardiometabolic disease prevention programs are cost effective [[29], [30], [31]]. Despite the limited evidence on cost-effectiveness, the substantial economic burden of cardiometabolic risk factors and disease in the US, in terms of both direct and indirect healthcare expenditure, supports the financial case for cardiovascular prevention programs [10,32,33].

**Fig. 1:**
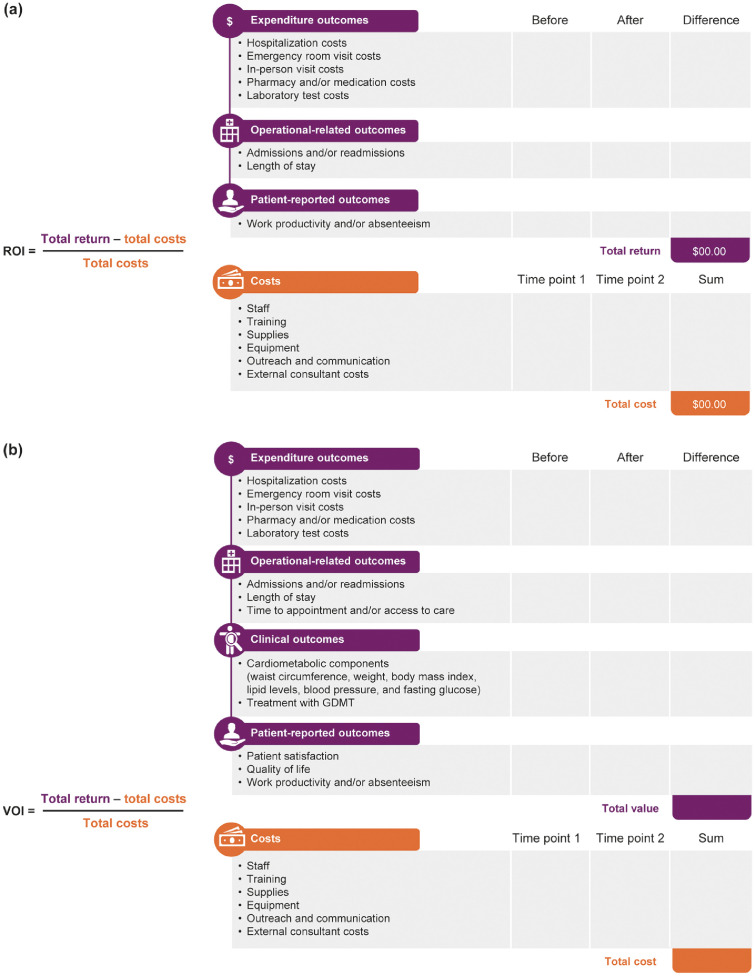
Proposed financial models for a CMC in the US demonstrating (a) ROI and (b) VOI.

CMC, cardiometabolic clinic; GDMT, guideline-directed medical therapy; ROI, return on investment; VOI, value on investment.

**Fig. 2:**
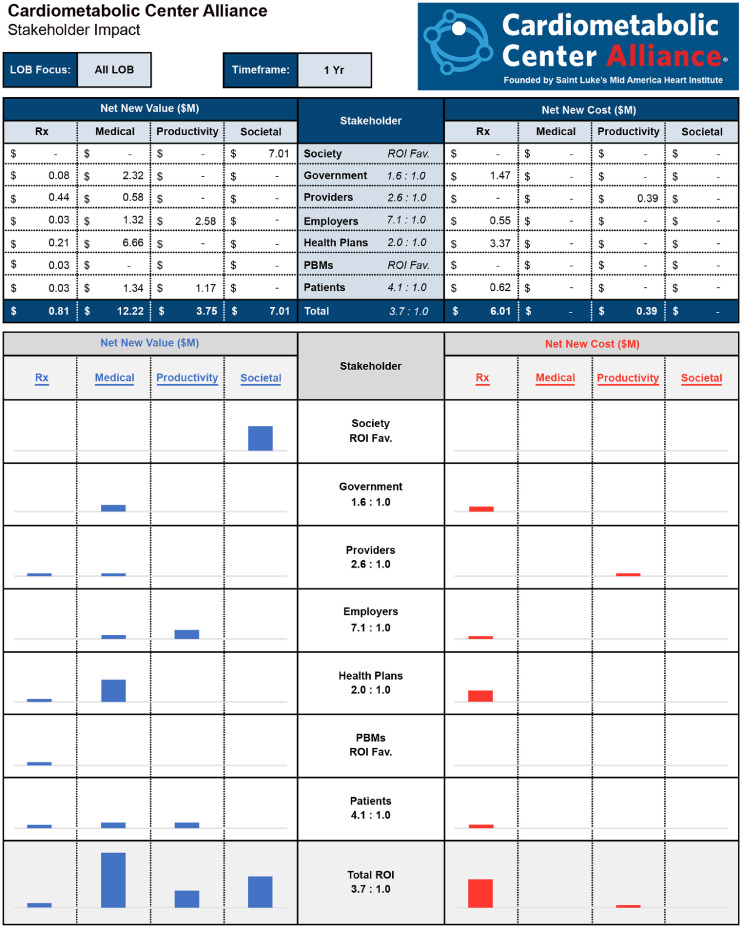
Example of a CMC ROI model built for the Cardiometabolic Center Alliance.

Developing a financial model that demonstrates the potential for a positive return on investment (ROI) to payers can help create payer medical policies that encourage reimbursement for and use of CMCs. Fig. 1**a** presents a proposed financial model for demonstrating ROI. A positive ROI (i.e., greater than or equal to 1) indicates that financial returns are greater than or equal to the investment costs; the more positive the ROI, the greater the financial gain [34]. As proposed in Fig. 1**a**, the financial model could include expenditure outcomes (e.g., hospitalization costs, emergency room and in-person visit costs, and pharmacy costs), operational-related outcomes (e.g., number of admissions), and patient-reported outcomes (e.g., work productivity and/or absenteeism). ROI models are relevant for CMCs due to the preventable events and measurable costs associated with cardiometabolic risk factors. This is particularly pertinent in the US, which has one of the highest per-patient costs globally for CVD, CKD, and T2D [[35], [36], [37], [38]], with healthcare expenditure on cardiometabolic risk factors and disease projected to increase in coming decades [27]. To ensure ROI models accurately capture a payer’s financial gains, they should be developed with a specific payer in mind, as the benefit depends on the costs and savings that payer incurs [28]. In addition to payer type, the time frame and patient population are also crucial to define, as these aspects can directly impact outcomes, as well as which outcomes are appropriate to include [39]. Studies examining ROI in cardiovascular prevention programs in the US demonstrate that longer time frames produce a greater ROI, as health outcomes improve and medical cost increases are mitigated [33,39]. Similarly, a high-risk patient population may generate greater financial returns, as changes in health outcomes and costs are greater than in low-risk groups [33,39]. As an example, Fig. 2 depicts a multidimensional ROI model that was built for the Cardiometabolic Center Alliance; in this model, the time period (i.e., 1, 3, 5, or 10 years), number of patient risk factors, and type of payer (e.g., commercial, Medicare, or Medicaid) can be adjusted to determine the corresponding ROI. However, ROI models may fail to accurately capture financial gains in CMCs for specific payers when patients switch insurance providers, which patients often do in the competitive multi-payer US healthcare system [28,40,41]. In addition, ROI models do not consider benefits beyond financial gains for payers. An alternative or complementary approach is developing a financial model that captures value on investment (VOI).

Stakeholder Impact Model. Cardiometabolic Center Alliance and Axene Health Partners. Reproduced with permission from Magwire M. (personal communication). CMC, cardiometabolic clinic; Fav, favorable; LOB, line of business; PBM, pharmacy benefit manager; ROI, return on investment; Rx, prescription; Yr, year.

A VOI model includes metrics related to monetary costs as well as patients’ health status and well-being to demonstrate the financial as well as broader societal benefits of a CMC [28,34]. As proposed in Fig. 1**b**, a VOI model could therefore include cost‑related outcomes but also outcomes that capture benefits beyond cost savings such as time to appointments, cardiometabolic components, patient satisfaction, and quality of life. Such models shift the focus towards the quality of care rather than the quantity. This is particularly relevant to CMCs where the aim is to deliver comprehensive and preventive care to patients. In recent decades in the US, value-based payment reforms have been implemented across cardiovascular care [42,43]. These payer models seek to replace one of the main healthcare reimbursement structures in the US: the “fee-for-service” model that reimburses payers and providers for the services delivered, i.e., focusing on quantity rather than quality [[41], [42], [43], [44]]. In contrast, value-based care models focus on reimbursing quality and cost [[42], [43], [44]].

These examples highlight key considerations for financial justification and also for the initial planning of a clinic. Creating a vision statement that defines the overall care objectives and target patient population are the first steps laid out by UNITE: Multidisciplinary Teams In Cardiometabolic Care, an initiative that has proposed a global CMC model that can be adapted to local needs [26]. Incorporating financial justification into these initial requirements reflects aspects specific to the US that should be considered in the development of a CMC.

### 2.2 Target patient population

Defining the high-level care objectives (i.e., primary and/or secondary prevention) and patient population is an important initial consideration, as they determine the care delivery and operational plans [26]. The national comprehensive care model from the US proposes a CMC that focuses on primary and secondary prevention of ASCVD and diabetes [24]. The clinic delivers value-based care, aimed at minimizing cost-intensive hospitalizations and healthcare expenditure [24]. It operates in an outpatient setting with coordinated care to reduce repeat diagnostic testing, patient visits to other clinics, and operating costs due to redundant workflow [24]. The model notes that multimorbid, complex patients with multiple risk factors would benefit most from the interdisciplinary approach of the clinic [24]. The model also suggests that children with cardiometabolic disease would be better suited for care from a pediatric endocrinologist [24]. A similar US model is the Cardiometabolic Center Alliance – Cardiometabolic Center of Excellence, which is focused on secondary prevention of cardiovascular risk in patients with T2D and CVD [45]. The model involves a multidisciplinary team that delivers patient-centered care to complex patients [45]. In a single-center study of this CMC, nurse navigators cross‑trained in both diseases were responsible for reviewing referrals, performing cardiometabolic assessments, and evaluating coverage for GDMT ahead of appointments [45]. A Cardiometabolic Center Advisory Committee provided guidance on patient selection, operating procedures, and care plans [45]. These models show that clinics can focus on different patient populations to deliver primary and/or secondary prevention, each requiring unique resources and strategies for developing a CMC.

In the US, numerous factors contribute to the decision to focus a CMC on patients with or without established disease, i.e., whether to focus on primary or secondary prevention, including the financial approach in place, local unmet needs, and staffing possibilities, Secondary prevention may be easier to justify to payers based on a higher likelihood of short-term financial returns. Patients with ASCVD, T2D, and CKD are at a higher risk of cardiovascular events and mortality than patients with risk factors for these diseases [[46], [47], [48]], meaning changes in outcomes (i.e., costs and savings) are likely to occur within a shorter time frame. In contrast, the financial benefits of primary prevention, even if equivalent to secondary prevention, may only become apparent over longer periods [49]. Hence, the financial approach is critical to defining the target patient population in the multi-payer system of the US. Geographic and rural-urban disparities in cardiovascular mortality and access to cardiologists and specialists in the US highlight the importance of considering the local unmet needs and staffing possibilities for where the clinic is planned [[50], [51], [52]]. A high prevalence of risk factors or disease, lack of access to specialists and preventive care, or gaps in existing clinics and referral pathways may influence the suitability of focusing on primary and/or secondary prevention. Finally, primary prevention may rely less on medical specialists in contrast to secondary prevention, meaning staffing possibilities including recruitment and retention, should be considered. Hence, clearly defining a target patient population is essential for the planning of a CMC.

### 2.3 Care delivery and operational plans: digital solutions and cost coverage evaluations

Subsequent steps involve developing a care delivery and operational plan. Key aspects include specifying referral sources and pathways and processes for patient intake, assessments, interventions (education, lifestyle, and pharmacologic), and follow-up, all of which inform operational elements (patient visits, staffing requirements, and clinic schedule) [26]. In the US, potential barriers to consider are low referral rates and limited accessibility to the CMC as a result of geographic and rural-urban disparities in relation to cardiovascular health and care [[50], [51], [52]]. Leveraging use of technology (e.g., telehealth, artificial intelligence, or mobile applications) can support referrals and access to care [44,53,54]. A modelling study of four cardiometabolic virtual-first programs from the US highlights the value of utilizing digital solutions for delivering remote services and encouraging patient engagement [54]. The study analyzed four cardiometabolic programs in which members received customized digital tools and resources that could be accessed via a computer or mobile device and access to an online peer support forum [54]. The study reported cumulative savings in medical expenditure over 5 years across the programs, as a result of improved health outcomes and delayed disease onset [54]. Furthermore, higher engagement levels correlated with greater improvements in clinical outcomes and medical cost savings [54]. In contrast, a systematic review of 13 studies evaluating mobile health applications to address cardiometabolic risk factors and health disparities in the US reported varying results in improvements in blood pressure control, physical activity levels, and nutritional intake [55]. However, most studies assessed feasibility, and varying results likely reflect methodological limitations, including short follow-up periods (10 studies <6 months), small sample sizes (8–533 patients), and a lack of control groups in 5 studies [55]. Tailoring digital solutions to the target patient population may help successful implementation. Providing training and education to primary care providers on cardiometabolic risk factors and the need for CMCs can further improve screening, identification, and referral of patients. Other important aspects to consider in the US are the costs of tests and treatment and insurance coverage. Incorporating evaluation of coverage and costs into the care plan can help ensure cost coverage where possible, which has shown to improve treatment adherence and cardiometabolic outcomes [56,57]. In summary, building a financial model, defining the patient population, implementing digital solutions, and integrating cost coverage evaluations are key elements for successfully developing a CMC in the US.

## 3 Structure of CMCs

In the global UNITE model, the proposed structure of a CMC involves a core team of key stakeholders, including a cardiology physician champion, administrative lead, and additional specialists where available [26]. National US CMC models showcase how multidisciplinary teams of primary care and specialist physicians, advanced practice providers, nurses, pharmacists, nutritionists, and health coaches, such as behavioral specialists and exercise physiologists, can be set-up and structured [24,45].

### 3.1 Comprehensive care model

A comprehensive care model from the US proposes setting up an outpatient clinic managed by two administrative personnel and a multidisciplinary team [24]. The proposed team includes cardiometabolic physicians, behavioral psychologists, nutritionists, certified diabetes care and education specialists, specialized rehabilitation physicians, and three cardiometabolic nurses [24]. In this model, cardiometabolic nurses play a central role in initial assessments, care plans, and follow‑up [24]. The cardiometabolic nurse triages new patients and conducts an initial comprehensive assessment and then consults with a cardiometabolic physician, who creates a personalized treatment plan for the patient [24]. The cardiometabolic nurse discusses the lab and imaging results with patients and coordinates follow-up appointments and, when necessary, referrals to other specialists [24]. Administrative personnel could take over scheduling appointments, but the nurse should provide continued care by providing lifestyle counselling and determining the need for follow-up appointments or referrals [24]. The model proposes that the cardiometabolic physician is responsible for cardiovascular imaging; management of lipids, glycemia, and obesity; assessment of chronic kidney disease and the associated cardiovascular risk through albuminuria measurement; and referrals to specialists such as metabolic surgeons [24]. If the patient requires medication(s), the cardiometabolic physician is responsible for prescribing, while a pharmacist manages medication plans, including medication access and adherence, and facilitates cost-saving interventions [24]. Managing follow-up care after cardiometabolic surgery should be a part of patients’ rehabilitation program [24]. Finally, the model proposes that the CMC should provide clinical training, including for students and residents, to train future cardiometabolic specialists [24].

### 3.2 Cardiometabolic center alliance – cardiometabolic center of excellence model

In a single-center study of the Cardiometabolic Center Alliance – Cardiometabolic Center of Excellence CMC, the multidisciplinary team comprised a nurse navigator, advanced practice practitioner, dietitian, and pharmacist in addition to a Cardiometabolic Center Advisory Committee that included endocrinologists and primary care physicians [45]. Nurse navigators cross-trained in the management of both T2D and CVD played a central role in the clinic [45]. Once the CMC was established, the nurse navigator screened patients before their initial visit [45]. At this visit, the patient was evaluated by a preventive cardiologist and, when appropriate, a certified diabetes educator, dietitian, and pharmacist [45]. The nurse navigator and advanced practice providers followed up with the patient using evidence- and guideline-based delivery of care [45].

### 3.3 Proposed structure

**Fig. 3:**
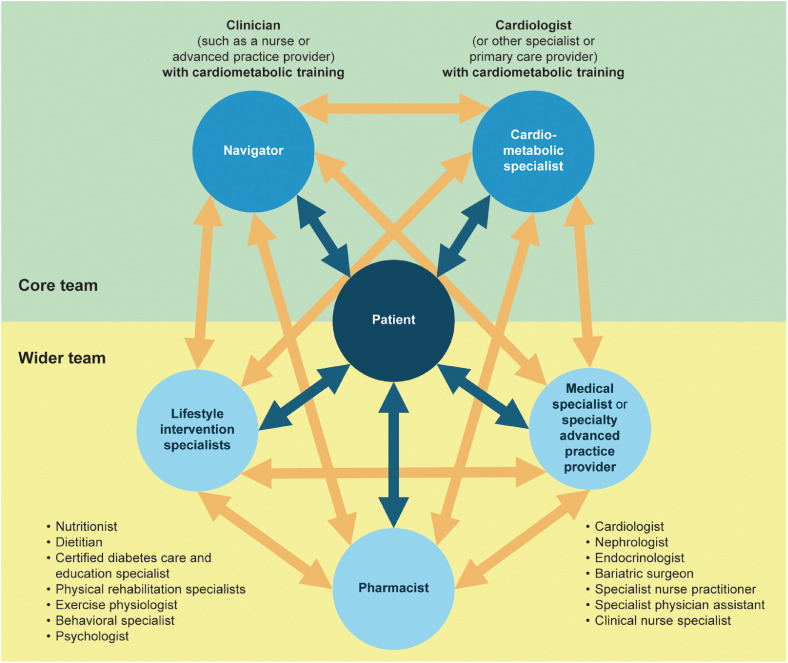
Central Illustration. Proposed structure of a CMC team, CMC, cardiometabolic clinic.

These models demonstrate how a CMC can be structured in the US, namely a core multidisciplinary team comprising a navigator and a specialist in cardiometabolic-related diseases (Fig. 3). A navigator role is crucial to the success of clinics, and it could be a nurse or other clinicians such as advanced practice providers, pharmacists, or certified diabetes educators [45]. The navigator role would involve screening patients, offering lifestyle counselling to patients, following up with patients to track progress, and referring patients to specialists when needed. The role of cardiometabolic specialist lead would ideally be taken on by a specialist in cardiology but could also be taken on by a specialist in endocrinology or nephrology or a primary care provider. The cardiometabolic specialist lead would be responsible for evaluating the patient, devising a comprehensive treatment plan, and prescribing treatments. However, knowledge gaps across the wide range of cardiometabolic conditions can make it challenging for a single individual to serve as a navigator or cardiometabolic lead. Training and educating the core team on cardiometabolic conditions (i.e., both ASCVD and T2D) are therefore essential, including assessment, management, and treatment across these disciplines [24,58]. In the US, there are online resources as well as several programs available for clinicians to gain specialized training in cardiometabolic health, such as a fellowship as well as Continuing Medical Education (CME) and non-CME courses [[59], [60], [61], [62]]. Once the clinic becomes more established, it could extend the services offered by additional providers and specialties and integrate these individuals into a wider team to support and fill any knowledge gaps of the core team and ensure patients receive guideline‑directed care (Fig. 3). A challenge in establishing a core and eventually wider team is the limited capacity and availability of specialists. Including advanced practice providers in the team can help streamline care as they can extend the capabilities of specialists [45].

## 4 Implementation of CMCs

### 4.1 Benefits to patients

Implementation of a CMC can ensure access to quality care, thereby improving patient outcomes and delivering positive ROI to payers and other healthcare stakeholders. Studies have shown an increased uptake of GDMTs after patients attend CMCs [45,[63], [64], [65]], with one study reporting uptake across racial subgroups [66]. In turn, several studies of CMCs have reported improvements in outcomes related to cardiometabolic components, including reductions in weight or waist circumference, cholesterol, blood pressure, and blood glucose [45,63,65,[67], [68], [69]]. Similarly, a real-world study in multi-ethnic patients with cardiometabolic risk factors found significant improvements in weight, body mass index, and blood pressure when patients were managed by a multidisciplinary team compared with primary care providers [70]. However, most studies of CMCs are non-randomized, cross-sectional analyses with small patient numbers. Studies with larger sample sizes over longer periods of time are lacking, as the practical implementation of CMCs has only recently begun to become more widespread. Studies with larger sample sizes are therefore needed and may also provide more robust insights into the cost effectiveness of CMCs. Nonetheless, evidence from multidisciplinary approaches brings to the forefront the importance of comprehensive cardiometabolic care and wider benefits to the patient. Studies of patients with T2D and coronary heart disease receiving multidisciplinary care have shown improvements in varying aspects of quality of life and patient satisfaction [71,72]. This highlights the opportunities of CMCs to improve the patient experience beyond strictly clinical outcomes by providing more comprehensive follow-up than in usual care.

### 4.2 Benefits to healthcare providers and the healthcare system

Wider advantages to patients and healthcare providers include convenience and improved communication. CMCs enable patients to see multiple healthcare providers at once and facilitate the coordination of care between patients and healthcare providers from different disciplines [58,73]. Streamlining care with a multidisciplinary team can benefit not only patients but also healthcare providers and the healthcare system by reducing redundant or unnecessary patient tests and hospitalizations, supporting good prescribing practice and lifestyle management, and minimizing costs [29,74]. By overseeing a patient’s treatment plan, referrals, and follow-up appointments, the core team of a CMC ensures a coordinated continuum of care and reduces the burden on healthcare providers otherwise operating in silos. Furthermore, CMCs offer the opportunity for core team members to gain specialized training in cardiometabolic health to build their knowledge in delivering evidence- and guideline-based care. This is particularly important given healthcare providers treating patients with these multimorbid conditions are faced with prescribing treatments outside of their specialty and changing specialist guideline recommendations and medication labels, which contribute to gaps in prescriptions of GDMTs [19,20,[75], [76], [77]]. Furthermore, physicians may not have the capacity, support, or training to deliver comprehensive and effective lifestyle counselling [18]. By incorporating specialists as part of the multidisciplinary team and managing referrals, a CMC further supports healthcare providers in following good prescribing practice and delivering high-quality care. Demonstrating a ROI for the health system supports further development and expansion of a CMC to justify allocation of additional resources, including personnel, training, equipment, supplies, and information systems [34]. Building a scalable clinic is essential to meet the likely growing demand of CMCs, with over one‑third of US adults receiving care for cardiometabolic risk factors and the prevalence of CKM syndrome continuing to rise [2,27,78].

### 4.3 Scalability of CMCs

Implementing a CMC with scalability in mind is crucial not only to accommodate the considerable and increasing number of patients with CKM syndrome but also to promote wider adoption and ensure long-term impact. ROI and VOI models can be used as evaluation tools to assess and justify scalability by demonstrating economic feasibility and value, respectively. However, a suitable tool or framework should be used to develop a scaling strategy; various healthcare‑specific tools are available that can be adapted to local needs and the target patient population [[79], [80], [81]]. Engaging different stakeholders (patients, providers, and payers) in the scalability strategy can help identify potential barriers and opportunities while ensuring that the scaling up process addresses the needs of all stakeholders and continues to promote social equity [[79], [80], [81]]. Organizing a team responsible for coordinating, monitoring, and adjusting scalability may also help a CMC transition from a pilot project to an established healthcare delivery approach [[79], [80], [81]]. Overall, scalability of a CMC remains crucial to delivering sustainable and societal impact that benefits patients, providers, and the wider healthcare system.

## 5 Conclusion

CMCs have the potential to reduce the clinical and financial burdens of cardiometabolic conditions on patients, healthcare providers, payers, and the wider healthcare system. Establishing a CMC in the US requires specific considerations due to national barriers, including the need for financial justification, digital solutions to improve accessibility, cost coverage plans, and cardiometabolic training. Building a foundational framework that can then be expanded is critical to implementing CMCs in the US for providing comprehensive patient care.

## Funding sources

Sponsorship for this manuscript was funded by Novo Nordisk Inc, which also reviewed the article for medical accuracy. The sponsor had no involvement in relation to the study design, collection, analysis and interpretation of data, writing of the report, and decision to submit the article for publication.

## Data statement

Data sharing is not applicable to this manuscript as no new data were generated or analyzed.

## CRediT authorship contribution statement

**Jodeanna Sweeney:** Writing – review & editing, Writing – original draft, Visualization, Methodology, Investigation, Conceptualization. **Jason Liu:** Writing – review & editing, Writing – original draft, Visualization, Methodology, Investigation, Conceptualization. **Gregory Warren:** Writing – review & editing, Writing – original draft, Visualization, Methodology, Investigation, Conceptualization. **Alexander J. Blood:** Writing – review & editing, Writing – original draft, Visualization, Methodology, Investigation, Conceptualization.

## Declaration of competing interest

The authors declare the following financial interests/personal relationships which may be considered as potential competing interests: All authors of the manuscript reports writing assistance was provided by Ashfield MedComms GmbH. Ashfield MedComms GmbH reports financial support was provided by Novo Nordisk Inc. Sponsorship for this manuscript reports financial support was provided by Novo Nordisk Inc. Jodeanna Sweeney reports a relationship with Novo Nordisk that includes: speaker fees. Jason Liu reports a relationship with Novartis that includes: consulting or advisory. Jason Liu reports a relationship with Alnylam that includes: consulting or advisory. Jason Liu reports a relationship with Novo Nordisk that includes: consulting or advisory. Alexander J. Blood reports a relationship with AstraZeneca that includes: funding grants. Alexander J. Blood reports a relationship with Boehringer Ingelheim that includes: funding grants. Alexander J. Blood reports a relationship with Eli Lilly that includes: funding grants. Alexander J. Blood reports a relationship with GE HealthCare that includes: funding grants. Alexander J. Blood reports a relationship with Merck that includes: funding grants. Alexander J. Blood reports a relationship with Novo Nordisk that includes: funding grants. Alexander J. Blood reports a relationship with Alnylam Pharma that includes: consulting or advisory. Alexander J. Blood reports a relationship with AstraZeneca that includes: consulting or advisory. Alexander J. Blood reports a relationship with Boehringer Ingelheim that includes: consulting or advisory. Alexander J. Blood reports a relationship with Color Health that includes: consulting or advisory. Alexander J. Blood reports a relationship with Corcept Therapeutics that includes: consulting or advisory. Alexander J. Blood reports a relationship with HelloHeart that includes: consulting or advisory. Alexander J. Blood reports a relationship with Medscape that includes: consulting or advisory. Alexander J. Blood reports a relationship with Milestone Therapeutics that includes: consulting or advisory. Alexander J. Blood reports a relationship with Nference Inc. that includes: consulting or advisory. Alexander J. Blood reports a relationship with NODE Health that includes: consulting or advisory. Alexander J. Blood reports a relationship with Novo Nordisk that includes: consulting or advisory. Alexander J. Blood reports a relationship with Walgreens Health that includes: consulting or advisory. Alexander J. Blood reports a relationship with Withings that includes: consulting or advisory. Alexander J. Blood reports a relationship with AIwithCare that includes: equity or stocks. Alexander J. Blood reports a relationship with Knownwell Health that includes: equity or stocks. Alexander J. Blood reports a relationship with Porter Health that includes: equity or stocks. Gregory Warren reports a relationship with AESARA that includes: consulting or advisory. Gregory Warren reports a relationship with Alnylam that includes: consulting or advisory. Gregory Warren reports a relationship with Arbutus that includes: consulting or advisory. Gregory Warren reports a relationship with Argenx that includes: consulting or advisory. Gregory Warren reports a relationship with Bausch Health that includes: consulting or advisory. Gregory Warren reports a relationship with Blueprint Medicines that includes: consulting or advisory. Gregory Warren reports a relationship with Cardiometabolic Centers Alliance that includes: consulting or advisory. Gregory Warren reports a relationship with Chiesi that includes: consulting or advisory. Gregory Warren reports a relationship with Collegium that includes: consulting or advisory. Gregory Warren reports a relationship with Free Market Health that includes: consulting or advisory. Gregory Warren reports a relationship with Genomic Life that includes: consulting or advisory. Gregory Warren reports a relationship with Gilead that includes: consulting or advisory. Gregory Warren reports a relationship with Lyceum Health that includes: consulting or advisory. Gregory Warren reports a relationship with Network of Advanced Specialty Healthcare that includes: consulting or advisory. Gregory Warren reports a relationship with Organon that includes: consulting or advisory. Gregory Warren reports a relationship with Novo Nordisk that includes: consulting or advisory. Gregory Warren reports a relationship with Piper Sandler that includes: consulting or advisory. Gregory Warren reports a relationship with Society of Actuaries Research Institute that includes: consulting or advisory. Gregory Warren reports a relationship with Solera Health that includes: consulting or advisory. Gregory Warren reports a relationship with Tempus that includes: consulting or advisory. If there are other authors, they declare that they have no known competing financial interests or personal relationships that could have appeared to influence the work reported in this paper.
